# Macroeconomic and distributive effects of increasing taxes in Spain

**DOI:** 10.1007/s13209-022-00269-5

**Published:** 2022-11-09

**Authors:** Luisa Fuster

**Affiliations:** Department of Economics, Universidad Carlos III de Madrid, Calle Madrid 126, 28903 Getafe, Madrid Spain

**Keywords:** Taxation, Fiscal pressure, Tax revenue, Entrepreneurship, Labor supply, Inequality, E2, E62, H24, H25, H30

## Abstract

I assess the macroeconomic and redistributive effects of tax reforms aimed at increasing tax revenue in Spain. To this end, I develop a theory of entrepreneurship that mimics key facts on the wealth and income distribution in Spain. I find two reforms that raise fiscal pressure in Spain to the average value among countries in the Euro area. The first reform involves doubling the average effective tax rate on labor and business income for all individuals whose income is above a threshold level. I find that this reform reduces the inequality in after-tax income, wealth, and consumption. However, it implies a substantial GDP reduction. The second reform increases the flat tax rate on consumption by fifteen percentage points. While this reform does not reduce long-run output, it does not decrease household inequality. All in all, the desirability of the two reforms depends on the government’s preferences for reducing inequality at the expense of aggregate output losses.

## Introduction

**Fig. 1:**
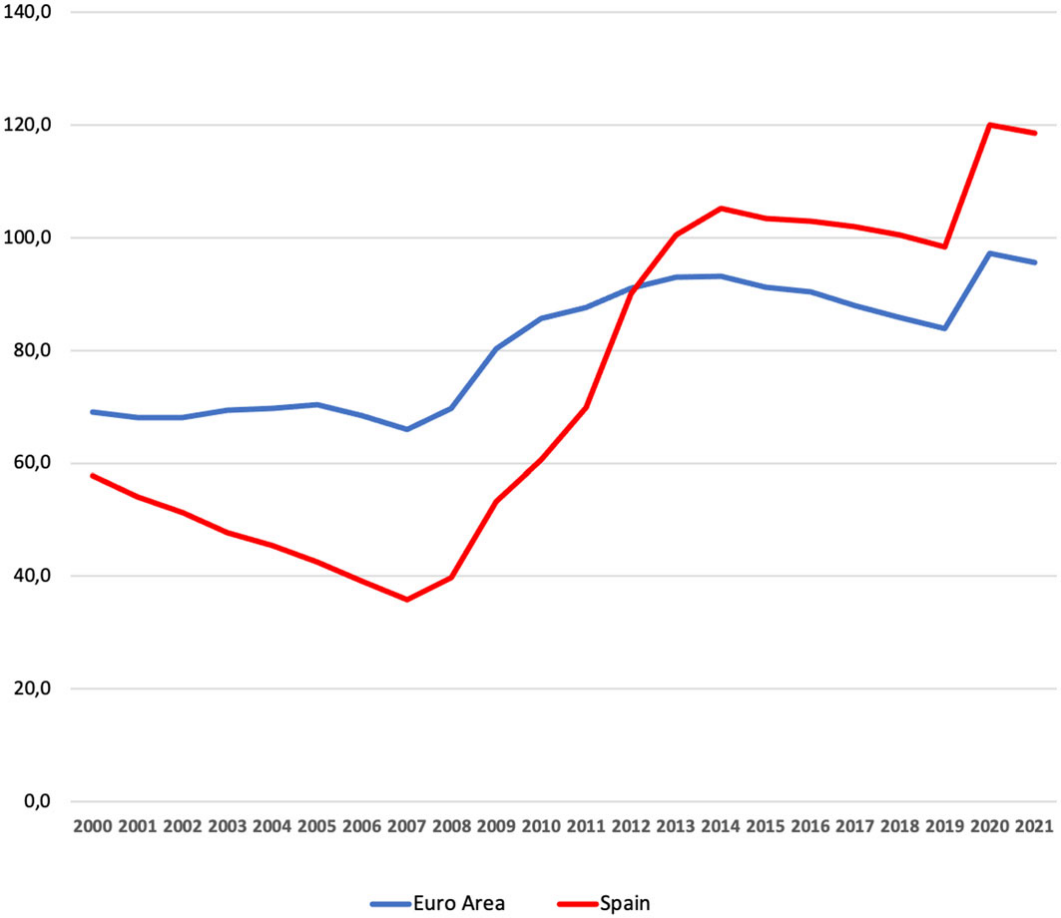
Government’s debt to GDP ratio (%): Spain and Euro area. Data: Government Finance Statistics (AGFS-Table S13), Eurostat 2022

Recent trends in the Spanish economy call into the sustainability of the welfare state. First, the aging of the Spanish population has increased the burden of the pay-as-you-go pension system. Second, the 2008 Great Recession has severely decreased tax revenue, increased inequality, and heightened the need to finance government transfers (such as unemployment insurance). Third, the recession and increase in expenditures caused by the Covid-19 pandemic have further complicated the government’s fiscal balance. As a result of these negative trends, in 2022, the Spanish government debt has reached a historical maximum of 120% of GDP (see Fig. 1). Since this level of debt is by now well-above the average debt to GDP ratio of the Euro area (95.6%) and of the EU-27 countries (88% of GDP), the need to raise government tax revenue is at the center of public policy debates in Spain.

**Fig. 2:**
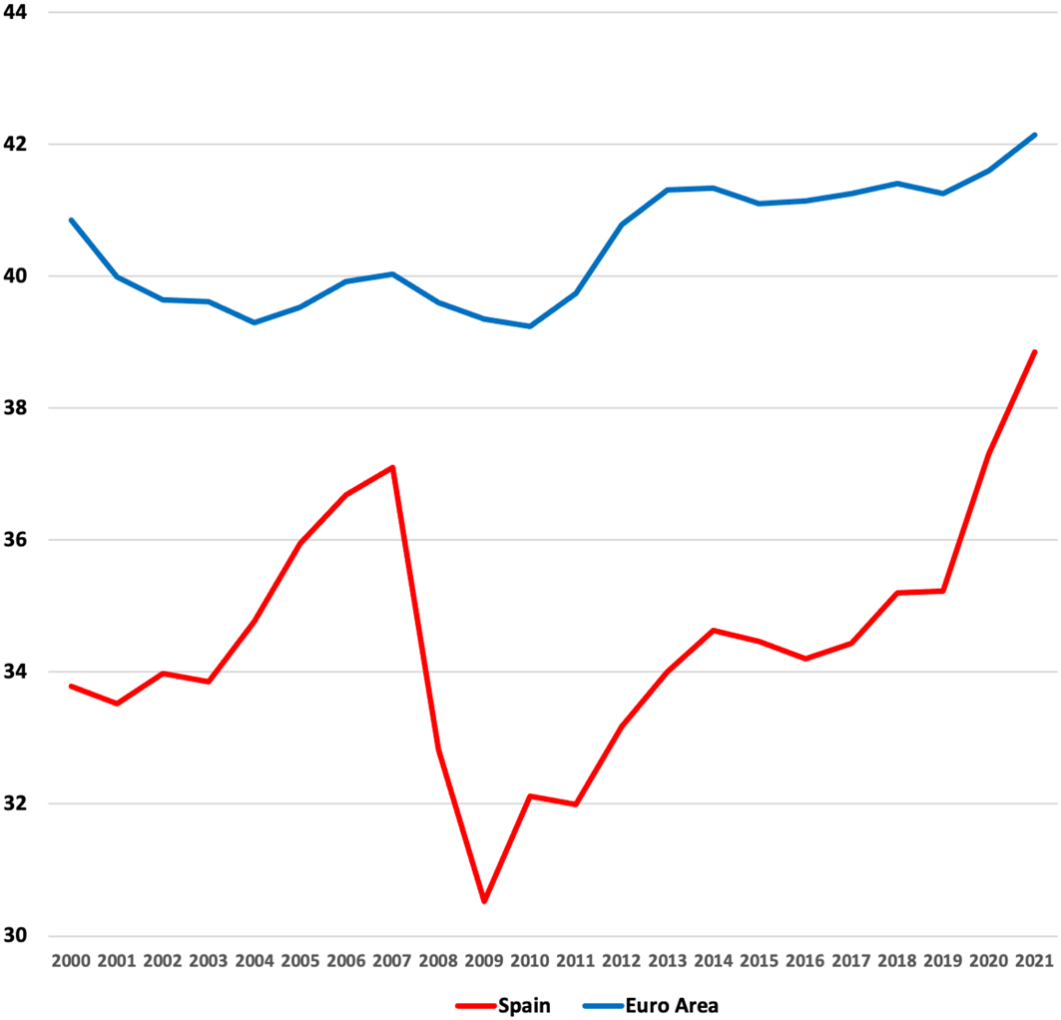
Total tax revenue to GDP ratio (%): Spain and Euro area. Data: Government Finance Statistics (AGFS-Table S13), Eurostat 2022

Advocates of raising tax revenue point to the fact that Spain collects low tax revenue in comparison with other countries in the Euro area (see Fig. 2). The AIREF (Independent Spanish Fiscal Authority) has recently recommended that the Spanish government increase the tax revenue through direct and indirect taxation. Moreover, in recent communication with the EU officials, the Spanish government has stated the following objectives: ‘It is necessary to increase fiscal pressure by 7.3 percentage points to close the gap with the Euro countries’ average, and to reform the tax system toward a more egalitarian, progressive, and fairer tax system’.^1^

The observations above motivate the following question: Can we increase the overall tax revenue in Spain and reduce income, wealth, and consumption inequality? To answer this question, I develop a computational model of the Spanish economy with heterogeneous agents and a rich tax structure. I assume that individuals make consumption, savings, and labor supply decisions. They also decide to be workers or entrepreneurs after observing a (stochastic) productivity realization affecting their productivity in each of the two occupations.^2^ Under these assumptions, some individuals have strong incentives to save in expectation of a high productivity entrepreneurial shock. The luckiest individuals frequently obtain high returns as entrepreneurs and accumulate wealth rapidly, allowing the model to account for the observed high concentration of wealth in Spain. While the poorest quintile of households in Spain owns zero or negative wealth, the top 80–100 quintile owns 70% of the wealth, and the top 1% of the richest households owns 20% of the Spanish wealth. The model is also consistent with the fact that entrepreneurs are over-represented among the richest households in Spain. In short, the theory developed accounts for key features of Spanish income and wealth distributions.

The theory captures essential aspects of the Spanish tax system. It features (nonlinear) taxes on personal income (the sum of labor and business income), capital income, inheritances, and wealth holdings. Corporate income and consumption expenditures are taxed at flat rates. The model mimics the sources of tax revenue of the Spanish government, with social security contributions accounting for 37% of the tax revenue, direct taxes on capital and labor accounting for 31%, and indirect taxation accounting for the remainder tax revenue.

**Fig. 3:**
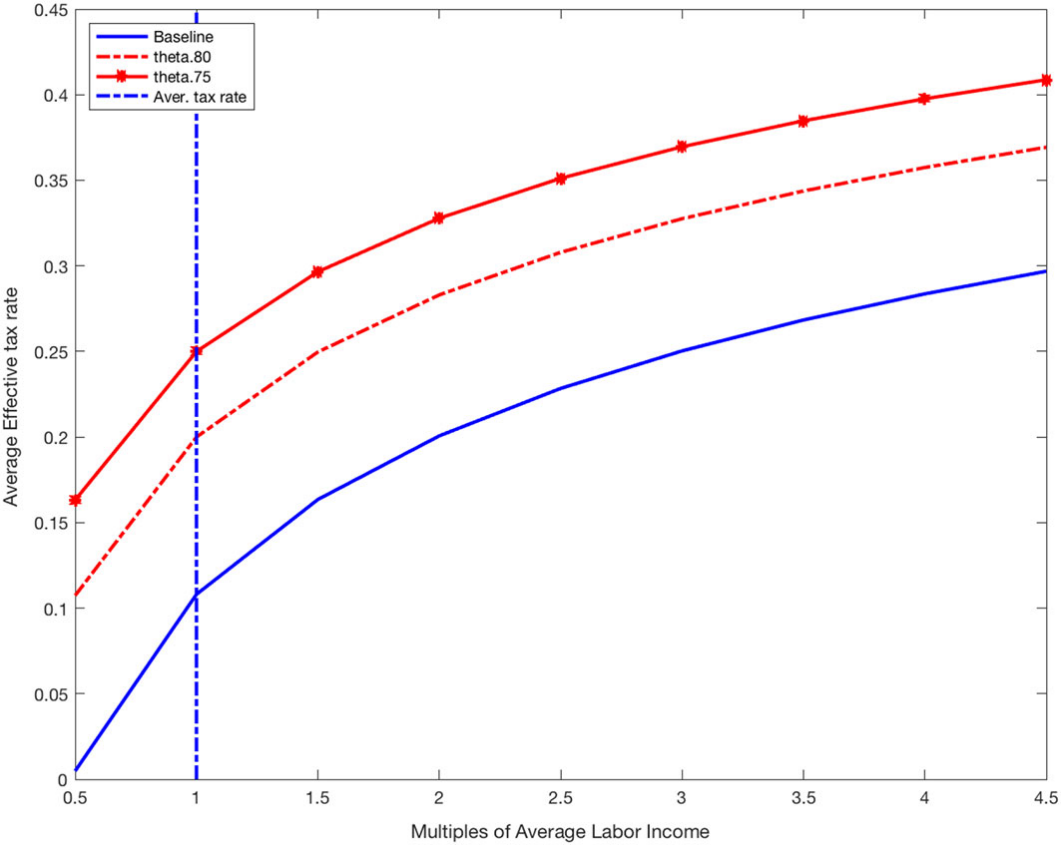
Average tax function: changing $\theta$

I model in detail the tax system in Spain. First, the personal income tax schedule is modeled following the standard approach of assuming an average (effective) tax function.^3^ In particular, personal income taxes (labor and business income taxes) in the model economy are set according to the tax function estimated by García-Miralles et al. (2019), who use data on tax records to estimate the tax function reported in Fig. 3. This function yields the average effective tax rate as a function of gross personal income in Spain. Two properties of the estimated tax function are worth highlighting. First, the estimated average tax rate is zero for individuals with personal income below a threshold value equal to half the mean income (11,175 euros in 2015). Second, the average tax rate on personal income increases for income above the estimated threshold. Specifically, the average tax rate equals 11% when income corresponds to the mean personal income in the economy (22,805 euros in 2015); it equals 18% for the 90th percentile of personal income (41,700 euros in 2015), and 28% for the 99th percentile of personal income (94,974 euros in 2015). The estimated average (effective) tax function underscores that personal income taxation plays an important role in redistributing income across households in Spain.

Having built a theory of household inequality in income and wealth in Spain that models the Spanish tax and transfer system, I simulate tax reforms that increase the overall tax revenue. I am interested in answering the following question: What are the long-run macroeconomic and distributive effects of fiscal reforms that increase the fiscal pressure (tax revenue per unit of GDP) to levels similar to the average among countries in the Euro area? I consider three main reforms.

The first reform increases the average (effective) tax rate on personal income (labor and business income) to all income levels above the exempt income level. I find that total tax revenue is maximized by a reform that increases the average effective tax rate from 11 to 24% at the mean income level, from 18 to 32% at the 90th income percentile, and from 28 to 40% at the 99th income percentile. The total tax revenue increases by 10% and fiscal pressure by 8 percentage points relative to the calibrated model economy. I find that the reform reduces inequality in after-tax income and consumption. This is partly explained by the fact that about 40% of the population does not pay any labor income taxes under the reform (individuals with income below 49% of the mean income in the economy do not pay personal income taxes). On the negative side, the reform reduces GDP by 8% and entrepreneurial output by 12%. Increasing tax rates on business income raises the opportunity cost of entrepreneurship. As a result, this reform decreases the number of entrepreneurs, the capital employed by them, and entrepreneurial output.

Second, I consider reforms designed to raise total tax revenue by increasing tax rates for income-rich and wealth-rich households. Specifically, I simulate reforms that increase the tax rates on interest income, wealth, and inheritances. Overall, I find that these reforms do not lead to a substantial increase in government revenue. The maximum tax revenue in these experiments is attained when the top marginal capital income tax rate is set to 60% (relative to the 20% marginal tax rate on capital income in the calibrated economy). The increase in the overall tax revenue is only 0.6%, which is an order of magnitude lower than the ones obtained by the first reform. Increasing tax rates for the income and wealth-rich households has negative effects on GDP and reduces social security contributions and the revenue from consumption taxation. On the positive side, the reform that maximizes the increase in total tax revenue reduces the Gini coefficient of consumption by 0.06. I also find that these reforms have positive effects on the output of entrepreneurs. As emphasized by Kitao (2008) and Guvenen et al. (2019), taxing interest income, wealth or inheritances more intensively decreases the opportunity cost of entrepreneurship.

Third, I explore the implications of increasing indirect taxes instead. In the baseline economy, the consumption tax is calibrated to match the effective consumption tax estimated by Bover et al. (2017) of 15%. I find that an increase in the consumption tax rate of 7 percentage points increases the overall tax revenue by an amount equal to that in the first reform. However, the two reforms considered have quite different macroeconomic and distributive effects. While GDP decreases by 8% under reform 1, GDP is not affected by the reform that increases the consumption tax rate. Moreover, unlike reform 1, the increase in consumption taxation does not reduce inequality in income, wealth, or consumption.

The increase in the consumption tax rate generates an income and a substitution effect on the labor supply. These effects cancel out because I assume logarithm utility on consumption (balanced growth path preferences). Moreover, savings decisions and the decision of whether to become an entrepreneur do not change when consumption taxes increase. As a result, the output of entrepreneurs and GDP are not affected by the increase in the consumption tax. All households decrease their consumption proportionally to the increase in the after-tax price of consumption and the inequality in the distribution of consumption does not change.

This paper is related to the quantitative macroeconomic literature studying how taxation affects entrepreneurship in the economy. For instance, (Cagetti and De Nardi 2009) quantify the impact of the elimination of estate taxation. Kitao (2008) focuses on how capital income taxation affects entrepreneurship. Meh (2005) quantifies the effects of eliminating the progressivity of income taxation in a model calibrated to the US data. Differently from these papers, I focus on quantifying the impact of tax reforms on the total tax revenue of the government. Similarly, (Bruggemann 2021) and (Imrohoroglu et al. 2018) quantify the impact of increasing the degree of progressivity of income taxation in the US economy on the government’s revenue. My paper extends their work by considering a larger set of tax instruments (wealth and inheritance taxation) and by considering reforms that increase the level of tax rates and not only the degree of progressivity of the tax code.^4^

There is a large macroeconomic literature that quantifies how much extra revenue the government can obtain by increasing the progressivity of income taxation in the US economy. Guner et al. (2016) calibrate a life cycle model with heterogeneous agents and a parametric function of the average (effective) tax rates on the income of households. They find that the maximum increase in government revenue is only 0.6% when the progressivity parameter of the tax function is increased. They conclude that increasing the marginal tax rate to 5% income earners from 21 to 42 is a more effective reform in terms of raising revenue (3.3%).^5^ Guner et al. (2020) quantify the impact of increasing the degree of progressivity of labor income taxation on the government’s revenue in a model calibrated to Spain. In the Spanish case, they conclude that there is little room for increasing the government’s revenue by making labor income taxes more progressive. Indeed, they find that the total revenue of the government decreases when the progressivity parameter is increased. Serrano-Puente (2020) analyzes the optimal degree of progressivity of income taxes in a dynastic framework calibrated to Spain. He concludes that the long-run aggregate welfare gains of increasing progressivity of labor income taxes are large.

The present paper is closely related to a literature that quantifies the effect of increasing income tax rates on the government’s revenue. Trabandt and Uhlig (2011) consider a representative agent model to quantify the Laffer Curve of the labor income tax for the USA and the UE14. More recently, (Holter et al. 2019) calibrate an heterogeneous agents household life cycle model to the US data and find that the peak of the Laffer curve is attained at an average labor income tax rate of 58%. This tax reform implies that revenue from labor income taxes increases by 59%. Erosa and González (2019) study the effect of alternative ways of taxing (corporate) capital income. Relatively to Holter et al. (2019) and Erosa and González (2019), I model entrepreneurship and the Spanish tax system and focus on the effects of tax reforms on overall tax revenue (not just on revenue from capital or labor income). Finally, this paper is also related to Conesa and Kehoe (2017), who quantify the impact of income taxation on labor supply for the Spanish economy in the spirit of Prescott (2004).

The paper is organized as follows: Section 2 documents some facts on entrepreneurship and wealth distribution in Spain. Section 3 presents the model economy. Section 4 describes the calibration methodology and the results of the calibration. Section 5 presents the results of the tax reforms, and Sect. 6 concludes.

## Wealth concentration and entrepreneurship in Spain

In this section, I document some facts on the inequality of wealth holdings in Spain that motivates modeling entrepreneurship for understanding the Spanish income and wealth distributions. The source of the wealth data is the Encuesta Financiera de las Familias (EFF) of the Bank of Spain. Using the 2017 wave, I compute the households’ net worth, which I define as total assets (financial, real state, and business) minus outstanding debt. I find that wealth inequality in Spain is substantial: The quintile 80–100 owns 70% of the wealth, and the top 1% most affluent household owns 21% of the wealth. Table 1 compares the share of wealth held by the richest households in Spain in 2017 with the ones reported by Cagetti and De Nardi (2006) for the USA. In Spain, the concentration of wealth at the top is substantial but not as large as in the US economy.

**Table 1:** Share of total wealth held by the richest households

	Top wealth percentile			
	1%	5%	10%	20%
Spain	21	46	58	70
US	30	54	67	81

Next, I study how entrepreneurship relates to wealth holdings in the data. To this end, I define an entrepreneur following (Cagetti and De Nardi 2006) as a head of households who answer yes to all of the following questions in the EFF of 2017: (1) Are you self-employed?; (2) Do you own a business?; (3) Do you or any family member actively manage your business? I find that in 2017, 7.75% of households were entrepreneurs in Spain. This compares to the finding of Cagetti and De Nardi (2006) for the US economy where 7% of households are entrepreneurs.

Table  2 reports the shares of entrepreneurs among the richest 1%, 5%, and 10% in Spain in 2017. It also compares these shares with the ones reported by Cagetti and De Nardi (2006) for the US economy. The table shows that entrepreneurs are concentrated among the wealthiest households in Spain. At the top 1% of the wealth distribution, 36% of households are entrepreneurs. The share of entrepreneurs is also large among the top 5% (27% of them are entrepreneurs) and among the top 10% richest households (15% of them are entrepreneurs).

**Table 2:** Percentage of entrepreneurs by wealth percentile

	Top wealth percentile		
	1%	5%	10%
Spain	36	27	15
US	54	39	32

Table  3 compares the wealth holdings across households with different occupations (of the head of the household) in Spain in 2017. The table shows that entrepreneurs are richer than workers and retirees. The mean net worth of an entrepreneur is 3.85 times that of a worker. The table also shows that the median wealth of an entrepreneur is 2.85 times that of a worker.

**Table 3:** Median and mean wealth (in thousand of Euros)

	Median	Mean
Whole population	118	252
Entrepreneurs	260	611
Workers	91	160
Retirees	232	444

To sum up, I highlight the following empirical facts:

1. Entrepreneurs represented 7.75% of the population in 2017 in Spain.
2. Among the top 1% richest wealth households in Spain, 36% are entrepreneurs. Among the 5% richest households in Spain, 27% are entrepreneurs.
3. Entrepreneurs are richer than workers. The ratio of mean wealth held by entrepreneurs relative to workers is 3.85, and the ratio of median wealth of entrepreneurs to median wealth of workers is 2.85.

The relationship between entrepreneurship and wealth concentration in the data motivates the development of an occupational choice decision model of the Spanish economy.

## The model

I build a general equilibrium exponential lifetime model where dynasties make consumption, savings, labor supply, and occupational choice decisions. The framework extends (Imrohoroglu et al. 2018) by modeling wealth and inheritance taxation and the income tax functions estimated by García-Miralles et al. (2019) for the Spanish economy.

### Demographics and preferences

Time is discrete. Each period, a new cohort of individuals enters the economy. Lifetime is divided into two stages: Youth and old-age. Initially, individuals are young and face a constant probability of becoming old. Young individuals are heterogeneous with respect to their labor productivity $z_w$ and managerial ability $z_e$, which evolve stochastically over time. Every period, they make an occupational choice decision regarding whether to be a worker or an entrepreneur. Differently, old individuals are not productive as workers but they are productive as entrepreneurs. This assumption implies that when a worker becomes old, the worker enters mandatorily into retirement. However, when a young entrepreneur becomes old, the entrepreneur can choose whether to retire or not. Retirement is an absorbing state during which individuals receive pension benefits. Every old individual faces a constant probability of dying. When an individual dies, her child enters the economy and inherits the family assets. Individuals are fully altruistic toward their descendants and maximize the discounted expected utility of their dynasty:

$$\begin{aligned} {{\,\mathrm{{\mathbb {E}}}\,}}\left[ \sum _{t=0}^\infty \beta ^t \left( \ln (c_t) -\phi \frac{h_t^{1+\gamma }}{1+\gamma } \right) \right] , \end{aligned}$$

where $c_t$ and $h_t$ denote consumption and hours of work at time *t*, $0<\beta <1$ is a time discount factor, $0<\phi$, and $0<\gamma$.^6^

### Production

At each date, there is one final good that can be produced by entrepreneurs or by firms in the corporate sector.

**Entrepreneurs** An entrepreneur with entrepreneurial ability $z_e$, labor productivity $z_w$, who works *h* hours, uses *k* units of capital, and hires *n* labor units (in efficient terms) produces an amount of output equal to

$$\begin{aligned} y_e=z_e(k^\alpha (n+z_w h)^{1-\alpha })^\nu \end{aligned}$$

where $0<\nu <1$, $0<\alpha <1$, and *n* denotes the efficient units of labor hired in the market. The capital *k* used by the entrepreneur is limited by the collateral constraint:

1 $$\begin{aligned} k \le \lambda a, \end{aligned}$$

where *a* denotes the assets owned by the entrepreneur and $\lambda \ge 1$ denotes the degree of financial frictions in the economy. The profits from business operation are the following:

2 $$\begin{aligned} \pi (a,z_w,z_e,h; r,w)&=\max _{k,n} \{ z_e(k^\alpha (n+z_w h)^{1-\alpha })^\nu -\delta k -r(k-a) - w n \} \nonumber \\&\text {subject to:} \nonumber \\&0\le k \le \lambda a, \nonumber \\&n\ge 0 \end{aligned}$$

where $0<\delta <1$ is the depreciation rate of capita, *w* is the wage rate per efficient unit of labor, *r* is the interest rate on assets, and the price of the produced good is normalized to 1.

**Corporate sector** The technology in the corporate sector is given by $F(K,L) = A K^{\alpha _c} L_c^{1-\alpha _c}$ where $0<\alpha _c<1$ and $0<A$. The specification of the production technology implies that the size distribution of firms in the corporate sector is irrelevant. Without loss of generality, I assume that there is a representative firm in the corporate sector that takes prices as given.

### Government programs

The government implements a tax and transfer program, consumes *G*, and administers a pay-as-you-go social security system.

#### Taxation

The government taxes consumption, capital income from the corporate sector, inheritances, wealth as well as household income. Household income is taxed differently depending on its source. The labor and business income are taxed with a progressive tax schedule, which differs from the tax schedule applied to household’s capital income. Moreover, capital income in the corporate sector is taxed at a flat rate.

**Household income taxation** Following (Guner et al. 2020), I assume that taxation of households’ income is determined by three functions: (i) $T_l (\cdot )$ determines the tax liabilities on labor income and business income; (ii) $T_k(\cdot )$ determines the tax liabilities on households’ interest income; (iii) $T_c(\cdot )$ determines tax credits to be deducted from the total tax liabilities on all the sources of household’s income.

Specifically, tax liabilities on labor income and business income are given by:

3 $$\begin{aligned} T_l(I_l)&= t_l(I_l/\bar{I_l}) \; I_l \; \text { and } \; t_l(I_l/\bar{I_l})=\left\{ \begin{array}{ll} 0 &{} \quad \text {if }I_l\le \hat{I_l} \\ 1-\theta (I_l/\bar{I_l})^{-\tau } &{} \quad \text {otherwise}. \end{array} \right. \end{aligned}$$

where $t_l(I_l/{\bar{I}})$ denotes the average tax rate as a function of the labor income of the household $I_l$ relative to the economy’s average labor income $\bar{I_I}$.^7^ Notice that the average tax rate equals zero if labor income is below a threshold level $\hat{I_l}$. Otherwise, the average tax rate is positive and increasing with the multiples of average labor income $I_l/{\bar{I}}$. The slope of this function is determined by the parameters $0<\theta$ and $0\le \tau$. In particular, $\tau$ controls the degree of progressivity of the tax function. For instance, if $\tau =0$, then the average tax is the constant $1-\theta$. The threshold level $\hat{I_l}$ allows the model to replicate the fact that a substantial fraction of households do not pay any labor income taxes in Spain. Tax liabilities on household capital income (interest income) are given by the function:

4 $$\begin{aligned} T_k(I_k)= t_k(I_k/\bar{I_k}) \; I_k \text { and } \; t_k(I_k/\bar{I_k})=\left\{ \begin{array}{ll} \eta _0+\eta _1 (I_k/\bar{I_k}) &{} \quad \text {if } I_k\le \ \hat{I_k} \\ \kappa &{} \quad \text {otherwise} \end{array} \right. \end{aligned}$$

The average capital income tax rate $t_k(I_k/\bar{I_k})$ is a function of the multiples of the economy’s average capital income $I_k/\bar{I_k}$ and a capital income threshold $\hat{I_k}$.

The households can deduct tax credits from their total tax liabilities on all sources of income. Tax credits are modeled with the function

5 $$\begin{aligned} T_c(I)= [\beta _0 +e^{\beta _1} e^{\beta _2 (I/{\bar{I}}) } (I/{\bar{I}})^{\beta _3}] I \end{aligned}$$

where $(I/{\bar{I}})$ denotes multiples of mean total income (labor, business, and capital income) and ${\bar{I}}$ denotes the mean income in the economy. The three functions just described were estimated by García-Miralles et al. (2019) using data from tax returns of Spanish households of 2015. I will use those estimates in the baseline model.

**Taxation of corporate income, inheritances, wealth, and consumption** I assume that capital income of the corporate sector is taxed at a flat rate $\tau _k$ and consumption at the rate $\tau _c$. Moreover, wealth is taxed at a flat rate $\tau _a$ above an exception level of wealth $\hat{I_a}$. I denote the average tax rate on wealth by

6 $$\begin{aligned} t_a(a)=\left\{ \begin{array}{ll} 0 &{} \quad \text {if } a < \hat{I_a} \\ \tau _a &{} \quad \text {otherwise} \end{array} \right. \end{aligned}$$

where *a* denotes the assets of the household. Inheritances are taxed according to a nonlinear tax schedule described in the calibration section. In what follows, I denote by $t_b(a)$ the average tax rate on inheritances, which is a function of the asset holdings at the time of death *a*.

#### Social security

The government administers a pay-as-you-go social security system. Retired individuals receive a pension $b(z_w)$ that depends on the last realization of their labor productivity. I assume that pensions are financed with a flat payroll tax $\tau _{ss}$ paid by workers. Because there is an upper bound to social security contributions in Spain, I assume that the social security tax is only applied to earnings up to 1.75 times the average earnings in the economy.^8^ In particular, social security contributions of a worker with earnings $w z_w h$ are given by:

7 $$\begin{aligned} T_{ss}(w z_w h)=\left\{ \begin{array}{ll} \tau _{ss} w z_w h, &{} \quad \text {if } w z_w h < 1.75 \bar{I_l} \\ \tau _{ss}1.75 \bar{I_l}, \ {} &{} \quad \text {otherwise } \end{array} \right. \end{aligned}$$

where $\tau {ss}$ denotes the contribution rate to social security and $\bar{I_l}$ denotes the economy’s mean earnings. On the other hand, a self-employed individual contributes a fixed amount to social security denoted by $T_{ss}^e$.

#### Government outlays

Following (Guner et al. 2019), I model mean-tested transfers as a linear function of household’s income *I*:

8 $$\begin{aligned} TR(I)=\left\{ \begin{array}{ll} g_0 {\bar{I}} &{} \quad \text {if } I=0 \\ g_1 {\bar{I}} +g_2 I &{} \quad \text {if } 0 < I \le 2.4 {\bar{I}} \end{array} \right. \end{aligned}$$

where ${\bar{I}}$ denotes the economy’s mean household income. In sum, aggregate tax revenue is used to finance pensions, mean-tested transfers, and the government expenditure *G*. For simplicity, I assume that the government has one budget constraint, which consolidates the budget constraints of the social security system and the treasury. Moreover, I abstract from government debt.

### The decision problem of households

Every period, households decide on consumption, labor supply, and savings. These decisions are taken under uncertainty regarding their productivity as workers and entrepreneurs, aging, and the timing of death. Since there are no insurance markets, households partially self-insure against the idiosyncratic shocks by building precautionary savings. I also assume that households cannot borrow in the asset market.

I use the dynamic programming language to describe the household problem and denote by $V^y$ and $V^o$ the value functions of a household when young and old, respectively. First, I consider the problem faced by old households. These households can be retired or be entrepreneurs. Retirement is an absorbing state in which the household collects pension benefits and decides how much to consume and save until death. If not retired, an old household decides whether to retire or not. I denote by $V^{o,e}$ the value of an old entrepreneur and by $V^{o,r}$ the value of a retired household. Then, the retirement decision of an old entrepreneur is represented by

9 $$\begin{aligned} V^o(a,z_w,z_e)= \max \{V^{o,r}(a,z_w),V^{o,e}(a,z_w,z_e)\}, \end{aligned}$$

where the value of a retired household is

10 $$\begin{aligned} V^{o,r} (a,z_w)&= \max _{c,a'} \left\{ \ln (c) + \beta \phi _o V^{o,r}(a',z_w) + \beta (1-\phi _o) {{\,\mathrm{{\mathbb {E}}}\,}}_{z_w^\prime ,z_e^\prime }[V^{y}(a'_{net},z_w^\prime ,z_e^\prime )] \right\} , \nonumber \\&\quad \text {subject to}, \nonumber \\&\quad (1+\tau _c) c + a' = \nonumber \\&\quad (1+r- t_a(a) )a + b(z_w) - T_l(b(z_w)) -T_k(r a) + \nonumber \\&\quad T_c(b(z_w)+r a)+TR(b(z_w)+r a), \\&\quad a'_{net}=a'(1-t_b(a')) \nonumber \\&\quad a' \ge 0. \nonumber \end{aligned}$$

The state of a retired household is given by the assets *a* and the labor productivity at the age of retirement $z_w$, which determines the pension received. A retired household survives to the next period with probability $\phi _o$ and dies with the complementary probability $1-\phi _o$. At death, the old household is replaced by a new household in the dynasty line who inherits the family assets net of bequest tax payments $a'_{net}$. The newly created household draws the entrepreneurial and labor skills ($z_w^\prime ,z_e^\prime$) from a fixed distribution (labor and entrepreneurial abilities of children and parents are not correlated). The value of an old entrepreneur with state $(a,z_w, z_e)$ is given by

11 $$\begin{aligned} V^{o,e} (a,z_w, z_e)&= \max _{c,h,a'} \left\{ \ln (c) -\phi \frac{h^{1+\gamma }}{1+\gamma }+\beta \phi _o {{\,\mathrm{{\mathbb {E}}}\,}}_{z_e^\prime }[V^o(a',z_w, z_e^\prime )/z_e] \right. \nonumber \\&\quad \left. + \beta (1-\phi _o) {{\,\mathrm{{\mathbb {E}}}\,}}_{z_e^\prime ,z_w^\prime } [V^{y}(a'_{net},z_w^\prime ,z_e^\prime )/z_e] \right\} , \nonumber \\&\quad \text {subject to:} \nonumber \\&\quad y^e=\pi (a,z_w, z_e, h)-\max \{0,r(a-k(a,z_w, z_e, h))\}, \nonumber \\&\quad y_a^e=\max \{0,r(a-k(a,z_w, z_e, h))\}, \nonumber \\&\quad (1+\tau _c) c + a' = \nonumber \\&\quad (1- t_a(a))a+y^e +y_a^e - T_l(y^e) - T_{ss}^e -T_k(y_a^e)\nonumber \\&\quad + T_c(y^e+y_a^e)+TR(y^e+y_a^e),\nonumber \\&\quad a'_{net}=a'(1-t_b(a'))\nonumber \\&\quad 0 \le h \le 1, \nonumber \\&\quad a' \ge 0, \end{aligned}$$

where $$\pi (a,z_w, z_e, h) $$ denotes the profits obtained by the entrepreneur and $$k(a,z_w, z_e, h) $$ the capital used in production in state $$(a,z_w, z_e)$$ and conditional on working *h* hours (see expression (2)). With probability $$\phi _o$$, the retired household survives and faces a retirement choice the next period. If the entrepreneurial household dies, I allow the entrepreneurial productivity of the new generation $$z_e^\prime $$ to be correlated with the previous households’ entrepreneurial ability in order to capture the idea that businesses can be inherited.

Second, I consider the decision problem of young households characterized by their state $(a,z_w, z_e)$. Every period, a young household makes an occupational choice decision represented by

12 $$\begin{aligned} V^y(a,z_w,z_e)=\max \{W^w(a,z_w,z_e), W^e(a, z_w, z_e)\} \end{aligned}$$

where $W^w(a,z_w,z_e)$ denotes the value of being a worker and $W^e(a, z_w, z_e)$ the value of being an entrepreneur. A young household that chooses to be a worker decides consumption (*c*), working time (*h*), and assets ($a'$) solving the problem:

13 $$\begin{aligned} W^{w} (a,z_w,z_e)&= \max _{c,h,a'} \left\{ \ln (c) -\phi \frac{h^{1+\gamma }}{1+\gamma } + \beta \phi _y {{\,\mathrm{{\mathbb {E}}}\,}}_{z_w^\prime ,z_e^\prime }[V^y(a',z_w^\prime ,z_e^\prime )/(z_w,z_e)]\right. \nonumber \\&\quad \left. + \beta (1-\phi _y) V^{o,r}(a',z_w) \right\} , \nonumber \\&\quad \text {subject to}, \nonumber \\&\quad (1+\tau _c) c + a' = (1+r- t_a(a))a + w z_w h - T_{ss}(w z_w h) - \nonumber \\&\quad T_l(w z_w h) -T_k(r a)+ T_c(w z_w h+ r a)+TR(w z_w h+ r a), \\&\quad 0\le h \le 1, \nonumber \\&\quad a' \ge 0, \nonumber \end{aligned}$$

where $\phi _y$ denotes the probability of not transiting to the old stage, in which case the household makes a draw of a new labor productivity $z_w^\prime$ and an entrepreneurial ability $z_e^\prime$ conditional on the current draws $(z_w,z_e)$. With probability $1-\phi _y$, the household becomes old and retires from the labor market the next period.

Alternatively, a young household can choose to be an entrepreneur and then solves the following problem:

14 $$\begin{aligned} W^{e} (a,z_w,z_e)&= \max _{c,h,a'} \left\{ \ln (c) -\phi \frac{h^{1+\gamma }}{1+\gamma } + \beta \phi _y {{\,\mathrm{{\mathbb {E}}}\,}}_{z_w^\prime ,z_e^\prime }[V^y(a',z_w^\prime ,z_e^\prime )/z_w,z_e]\right. \nonumber \\&\quad \left. + \beta (1-\phi _y) {{\,\mathrm{{\mathbb {E}}}\,}}_{z_e^\prime } [V^{o}(a',z_w,z_e^\prime )/z_e] \right\} , \nonumber \\&\quad \text {subject to}, \nonumber \\&\quad y^e=\pi (a,z_w, z_e, h)-\max \{0,r(a-k(a,z_w, z_e, h))\} \nonumber \\&\quad y_a^e=\max \{0,r*(a-k(a,z_w, z_e, h))\},\nonumber \\&\quad (1+\tau _c) c + a' = \nonumber \\&\quad (1- t_a(a))a+ y_e+y_a^e - T_{ss}^e - T_l(y^e) \nonumber \\&\quad -T_k(y_a^e)+ T_c(y^e+y_a^e)+TR(y^e+y_a^e) \nonumber \\&\quad 0\le h \le 1, \nonumber \\&\quad a' \ge 0, \end{aligned}$$

where $\pi (a,z_w, z_e, h)$ denotes the profits obtained by an entrepreneur and $k(a,z_w, z_e, h)$ the capital used in production given the state $(a,z_w, z_e)$ and conditional on working *h* hours (see expression (2)). Note that the entrepreneur becomes old and faces a retirement decision with probability $1-\phi _y$, which will be taken after making a new draw of the entrepreneurial idea $z_e^\prime$.

#### The decision problem of corporate firms

If aggregate output of the corporate sector is $Y_c$, the production plan of the representative firm in the corporate sector solves the following cost minimization problem:

15 $$\begin{aligned}&\min _{K_c, N_c} (r (1+\tau _k)+ \delta ) K_c+ w N_c \nonumber \\&\text {subject to,} \nonumber \\&A K_c^{\alpha _c} N_c^{1-\alpha _c} \ge Y_c \end{aligned}$$

Note that the user cost of capital depends on the equilibrium interest rate *r*, the corporate tax rate $\tau _k$, and the depreciation rate of capital $\delta$. I assume that the corporate income tax only applies to the return on capital net of depreciation.

### Competitive equilibrium

I focus on stationary competitive equilibrium. Denote by $$\Gamma (s)$$ the invariant measure of young and old households across states $$s= (a, z_w, z_e, j)$$, where state $$j=\{y,r, n_r\}$$ indicates whether the household is young (y), retired (r), and old but not-retired ($$n_r$$). In general, households make decisions on consumption (*c*), savings ($$a^\prime $$), hours of work (*h*), occupational choice (represented by indicator functions $$I_e$$ and $$I_w$$), and retirement $$(I_r)$$. Denote these policy functions by $$(c(s), a^\prime (s), h(s), I_e(s), I_r(s))$$. Furthermore, I denote the policy functions giving the capital and labor services hired by entrepreneurs by $$ (k^d(s),n^d(s))$$, where it should be understood that $$(k^d(s),n^d(s))=(0,0)$$ among non-entrepreneurial households. Using this compact notation, a stationary equilibrium is given by a collection of value functions and policy functions, corporate sector aggregates $$(K_c,N_c, Y_c)$$ and prices (*w*, *r*) such that:

1. The value functions of households satisfy the Bellman equations (10), (11), (13), and (14).
2. Policy functions are optimal (maximize the RHS of the Bellman equations).
3. Labor, capital, and goods markets clear:
  16 $$\begin{aligned}&\int _{\{s: I_w(s)=1\}} h(s) z_w(s) \Gamma ({\textrm{d}}s) = \int _{\{s: I_e(s)=1\}} n^d(s) \Gamma ({\textrm{d}}s)+ N_c, \end{aligned}$$
  17 $$\begin{aligned}&\int a(s) \Gamma ({\textrm{d}}s) = \int _{\{s: I_e(s)=1\}} k^d(s) \Gamma ({\textrm{d}}s) + K_c,\end{aligned}$$
  18 $$\begin{aligned}&\int (c(s) + \delta k^d(s)) \Gamma ({\textrm{d}}s) + \delta K_c + G \nonumber \\&\quad = \int _{\{s: I_e(s)=1\}} z_e(s) (k^d(s))^{\alpha \nu } (l^d(s))^{(1-\alpha ) \nu } \Gamma ({\textrm{d}}s) + Y_c, \end{aligned}$$
  where $$Y_c= F(K_c,N_c)$$ is the output of the corporate sector and, with a slight abuse of notation, I denote by $$z_w(s) $$ the value of labor productivity of a household in state $$s= (a, z_w, z_e, j)$$.
4. The government’s budget is balanced at every period.
5. The distribution of agents across states $\Gamma (s)$ is invariant, that is, it reproduces itself according to a given transition function.

To write the government’s budget constraint using a compact notation, I define $y_w(s)= z_w(s) h(s) w$ as the labor earnings of a worker in state *s* (which is only relevant in states for which $I_w(s)=1$). Similarly, I define $y_e(s)$ as the business income of an entrepreneur and $y_e^a(s)$ the interest income of an entrepreneur in state *s* (only relevant in states such that $I_e(s)=1$). Then, the government outlays are given by:

19 $$\begin{aligned} \text {Outlays}&= G+ \int _{\{s: I_r(s)=1\}} b(z_w) \Gamma ({\textrm{d}}s) \nonumber \\&\quad + \int _{s}T_c\left( (y_e(s)+y_e^a(s)) I_e(s) +(y_w(s)+ra) I_w(s)\right) \Gamma ({\textrm{d}}s) \nonumber \\&\quad + \int _{s} \left( TR\left( y_e(s)+y_e^a(s) \right) I_e(s) +TR\left( y_w(s)+ra\right) I_w(s) \right) \Gamma ({\textrm{d}}s) \end{aligned}$$

where I have added government purchases of goods, payments to retired households, tax credits, and transfers. The government tax revenue is given by

20 $$\begin{aligned} \text {Revenues}&= \int _s [ \tau ^c c(s) + t_a(a/I_a) a(s) + T_{ss} (y_w(s)) I_w(s) + T_{ss}^e I_e(s) ] \Gamma ({\textrm{d}}s) \nonumber \\&\quad + \int _s [ T_l(y_w(s) )I_w(s) +T_l(y_e(s) )I_e(s) +T_l(b(z_w) )I_r(s) ] \Gamma ({\textrm{d}}s) \nonumber \\&\quad + \int _s [ T_k(y_e^a(s)) (I_e(s)+I_{nr}(s)) +T_k(r a)( I_w(s)+I_r(s)) ] \Gamma ({\textrm{d}}s) \nonumber \\&\quad + \tau _k r K_c + (1-\phi _o) \int _{\{s: I_r(s)=1 \text { or } I_{nr}(s)=1\}} a'(s) \tau _b(a'(s)) \Gamma ({\textrm{d}}s), \end{aligned}$$

where the first line above adds the tax revenue on consumption taxes, wealth taxes, and social security contributions by workers and entrepreneurs. The second line adds income taxes on labor and business income. The third line adds taxes on interest income of entrepreneurs, workers, and retired individuals. The fourth line adds tax revenue from corporate income and bequests (inheritances). To compute the latter, I have made use of the fact that old households, whether retired or not, die with probability $(1-\phi _o)$.

To compute steady-state equilibrium, I guess prices (*w*, *r*) and the income thresholds that define tax rates. Then, I solve the household problem and the problem of the representative firm in the corporate sector. I aggregate decisions over all households using the invariant measured of households $\Gamma$ implied by the optimal policy functions and stochastic processes on labor productivity and entrepreneurial skills. The value of government purchases is the one that equates government outlays to tax revenue. I then check if the labor and asset markets clear. If not, I make a new guess on prices and income thresholds until convergence.

## Calibration

A subset of model parameters are fixed exogenously (Tables 4, 5, and 6) while other parameters are calibrated by solving the model with the objective of matching moments in the data (Table 7). In what follows, I describe the choice of functional forms and the calibration methodology.

### Demographics and preferences

The model period is equivalent to one year. I assume that individuals are born at the age of 25. The probability of becoming old $(1-\phi _Y)$ is chosen so that, on average, individuals become old at the age of 65. The probability of dying $(1-\phi _O)$ is set to match that in Spain, about one-third of the adult population were individuals older than 65 in 2017.^9^ This choice of $\phi _o$ is consistent with an expected lifetime of 85.

I assume the period utility function $u(c,h)=\ln (c) -\phi \frac{h^{1+\gamma }}{1+\gamma }$. This utility function is consistent with balanced growth and with evidence from panel data that, conditional on education, mean lifetime hours of work do not vary across the distribution of lifetime wages (see Erosa et al. 2016). The parameter $\gamma$ is set so that the Frisch elasticity of labor supply equals $1/\gamma =0.5$ as in Guner et al. (2020), which facilitates the comparability with their findings. The discount factor of time $\beta$ and the weight of the disutility of work $\phi$ will be calibrated endogenously.

### Production

I assume a capital share $\alpha =\alpha _c= 1/3$, a span of control parameter $\nu =0.88$, and a depreciation rate $\delta =0.06$, which are standard values in the literature (see, for instance, (Bassetto et al. 2015; Allub and Erosa 2019), and (Erosa et al. 2021). The TFP parameter (A) in the corporate sector is normalized to 1.

The capital used by the entrepreneur is limited by the collateral constraint $k \le \lambda a$. I set $\lambda =1.5$ meaning that entrepreneurs can borrow up to 50% of the value of their wealth. This assumption is roughly consistent with the average tangibility of firms in Spain in 2010. In particular, (García-Posada et al. 2014) find an average tangibility of 148% for micro-firms and 154% for other firms using firm-level data from the OECD-Orbis Data Base.

In the model, the entrepreneurial ability $z_e$ follows the AR(1) process:

21 $$\begin{aligned} \ln (z_{e,t})=(1-\rho _{e}) \ln (\mu _e) +\rho _{e} \ln (z_{e,t-1}) +\epsilon _t, \epsilon _t \sim N(0,\sigma _{e}^2) \end{aligned}$$

I set the coefficient of correlation $\rho _{e}=0.9$, which is in the range of values assumed in the literature (see, for instance Allub and Erosa 2019 and Wellschmied and Yurdagul 2021). The parameters ($\mu _e,\sigma _{\epsilon }$) are calibrated endogenously to match the fraction of entrepreneurs in the population and the share of wealth held by the top 1% richest households in Spain in 2017. I discretize the AR(1) process of entrepreneurial ability using the Tauchen method (see Tauchen 1986).

### Labor productivity process

The logarithm of labor productivity $\ln (z_w)$ is specified to follow an AR(1) process:

22 $$\begin{aligned} \ln (z_{w,t})= \rho _{w} \ln (z_{w,t-1}) +\eta _t, \eta _t \sim N(0,\sigma _{w}^2) \end{aligned}$$

I set $\rho _w=0.96$ which is a standard value in the literature. The standard deviation of the innovations to labor productivity $\sigma _w$ is chosen to match a Gini coefficient of labor earnings 0.36 in Spain in 2017 (Encuesta Financiera de las Familias). I use the Tauchen method to discretize the AR(1) process of the labor productivity in a discrete grid (size 10).

### Tax functions and parameters

Table 4 reports the values of parameters of the income tax functions (3) and (4) and the tax-credit function (5) which were estimated by García-Miralles et al. (2019) using data on tax records of 2015. The mean-tested transfers function (8) is parametrized as in Guner et al. (2019) and I set $g_0=0.04$, $g_1=0.024$, and $g_2=0.01$.

**Table 4:** Income tax functions

Labor income tax	Capital income tax	Tax credits
$\theta =0.8919$	$\eta _0=0.1272$	$\beta _0=0.0085$
$\tau =0.1581$	$\eta _1=0.0057$	$\beta _1=12.5683$
$\hat{I_l}\big / \bar{I_l}=0.49$	$\kappa =0.2018$	$\beta _2=-17.5032$
	$\hat{I_k}\big /\bar{I_k} =13.14$	$\beta _3=14.4012$

The social security tax rate is fixed to 28%. It applies to earnings up to a maximum taxable level equal to 1.75 times the average earnings in Spain (monthly earnings of 3600 euros in 2015). I parameterize the contribution to social security by a self-employed individual, $T_{ss}$, so that the aggregate contributions by self-employed workers equals 12% of the total social security contributions. The pension formula assumed is $b(z_w)=rr(z_w){\bar{I}}_{z_w}$, where ${\bar{I}}_{z_w}$ denotes the mean earnings of workers with labor productivity $z_w$. The replacement rates $rr(z_w)$, for each of the 10 grid points of the labor productivity $z_w$, are set to match moments of the distribution of pensions in Spain in 2017.^10^

In 2015, the level of wealth exempt from taxation was 700,000 euros (roughly 30 times the average individual income in the economy). I assume that $\tau _a =0.42\%$, which corresponds to the average effective tax rate across Spanish regions in 2015.^11^

The inheritance tax is characterized by a nonlinear schedule and multiple tax credits that vary across regions. De la Fuente (2018) provides an indicator that summarizes the different tax credits applied in each region (see Table 12 in De la Fuente (2018)). The average of this indicator (bonus rate) across all regions was 46.2% in 2016. This estimate implies that on average, the inheritance taxes paid were about 58% of the quota implied by the statutory tax schedule reported in Table 5.

Regarding consumption taxation. I set $\tau _c= 0.15$ since (Bover et al. 2017) find that the effective consumption tax rate in Spain is about 15% . Finally, I set $\tau _k=0.25$, which was the statutory corporate income tax rate in Spain in 2015.

**Table 5:** Inheritances: statutory tax schedule

Bequest Brackets (thousand Euros)															
0–8	8–16	16–24	24–32	32–40	40–48	48–56	56–64	64–72	72–80	80–130	130–160	160–240	240–400	400–800	+800
Tax rates															
0.0765	0.085	0.0935	0.102	0.1105	0.119	0.1275	0.136	0.1445	0.1530	0.1615	0.187	0.2125	0.255	0.2975	0.34

**Table 6:** Parameters calibrated exogenously

Parameters	Explanation	Value
*Demographics*		
$1-\phi _Y$	Prob. becoming old	1/40
$1-\phi _O$	Prob. death	1/20
*Preferences*		
$\gamma$	Inverse Frisch elasticity labor supply	2.0
*Technology*		
$\nu$	Span Control	0.88
$\alpha$	Capital share	0.33
$\delta$	Depreciation rate capital	0.06
*A*	Total factor productivity corporate sector	1.0
*Entrepreneurial ability and labor productivity*		
$\rho _{e}$	Entrepreneurial ability AR(1) process	0.90
$\rho _{w}$	Labor productivity AR(1) process	0.96
*Taxation*		
$\tau _c$	Consumption tax rate	0.15
$\tau _w$	Wealth tax rate	0.0042
$\tau _k$	Corporate income tax rate	0.25
$\tau _{ss}$	Payroll tax rate	0.28

### Parameter values set by solving the model

It remains for the values for $\phi , \beta$, $\sigma _{w}$ , $\mu _e$, and $\sigma _e$ to be chosen. I search for the values of these five parameters that minimize the sum of the square deviations between selected model statistics and their data counterparts. It is useful to discuss how each of the parameters connects with moments in the data targeted. The discount factor, $\beta$, affects the equilibrium rate of return on capital and hence the economy’s *K*/*Y* ratio. The weight of labor disutility $\phi$ determines mean hours of work in the economy. The variance of the labor productivity shock will be chosen to match the Gini coefficient of earnings in Spain in 2017. The parameter $\mu _e$ of the stochastic process of entrepreneurial log-ability affects the fraction of entrepreneurs in the population. The standard deviation of the innovation of the AR(1) process of the entrepreneurial ability affects the share of wealth held by the top 1% and 10% percentiles of the wealth distribution. Table  7 reports the values of the calibrated parameters and shows that the model matches well the calibration targets.

**Table 7:** Parameters estimated and calibration results

Parameters	Explanation	Value	Statistic	Data	Model
$\beta$	Time discount factor	0.9688	K/Y	3	3
$\phi$	Weight of labor disutility	11.9124	%Mean working time	38	39
$\sigma _{w}$	SD log labor productivity	0.532	% Entrepreneurs in pop.	7.0	6.8
$\mu _e$	Entrepreneurial ability process	0.0000026	Gini Earnings	0.37	0.36
$\sigma _e$	SD log entrepreneurial ability	0.3475	% Share wealth at top 10%	58.3	61
			% Share wealth at top 1%	20.8	21

### Implications of the model for non-targeted statistics

Table 8 shows that the model mimics the fact that entrepreneurship is concentrated at the top percentiles of the wealth distribution. For instance, among the top 10%, top 5%, and top 1% wealth richest households, the fraction of entrepreneurs is 15%, 27%, and 36% in the baseline model. These numbers compare very well with their data counterpart of 19.7%, 25%, and 37% (EFF 2017), respectively. Table 8 also reports the mean net worth of entrepreneurs and workers. The ratio of the mean net worth of entrepreneurs to workers equals 3.8 in the baseline model, while it is about 3 in the data (EFF 2017).

**Table 8:** Entrepreneurship and wealth concentration

Statistic	Data	Model
%Entrep. at the Top 10% wealth	15	19.7
%Entrep. at the Top 5% wealth	27	25
%Entrep. at the Top 1% wealth	36	37
Ratio median Net-worth Entrep. to workers	2.85	4.1
Ratio mean Net-worth Entrep. to workers	3.81	3.1

The calibration of the baseline model targeted the shares of wealth held at the top 1% and top 10% of the distribution of wealth. Table 9 shows other moments of the wealth distribution in the baseline model and compares them to the statistics in the data (EFF, 2017). The baseline model reproduces the fact that the bottom quintile does not hold positive wealth in Spain in 2017. It understates the wealth holdings of the 20–40 quintile (1% in the baseline model vs 4% in the data), while it matches the wealth holdings of the 60–80 quintile well (15% in the model vs 18% in the data). In summary, the calibrated model fits the wealth holdings of the top 90 percentile, and of quintiles 0–20 and 60–80. However, the model understates the wealth holdings of the quintiles 20–40 and 40–60.

**Table 9:** Wealth distribution: model versus data

	Quintiles					Top			Bottom
	0–20	20–40	40–60	60–80	80–100	90–95	95–99	99–100	0–10
Data	$-$ 0.5	3.8	9.3	17.9	69.6	13.0	24.6	20.8	$-$ 0.6
Baseline	0	1.1	5.1	15.3	78.5	16	24	21	0.0

Next, I focus on the implications of the model regarding the distribution of income. Table 10 compares the share of income owned by each quintile of the distribution of income across the baseline economy and the data (EFF 2017). The baseline model reproduces the income shares across the quintiles quite well, at the top 90 percentile, and the bottom 10 percentile. Since entrepreneurship generates a high concentration of wealth at the top of the distribution, the income distribution also shows concentration of income at the top. For instance, the share of income of the quintile 80–100 is 50.6% in the baseline model, which compares well to the share of 48.4% of income in the data.

**Table 10:** Income distribution: model versus data

	Quintiles					Top			Bottom
	0–20	20–40	40–60	60–80	80–100	90–95	95–99	99–100	0–10
Data	5.1	9.7	14.7	22.1	48.4	10.8	15.6	8.9	2.0
Baseline	5.9	9.3	14	20.4	50.6	11.8	13.1	11.0	2.2

Table 11 reports the shares of the personal income tax payments by income quintiles. The baseline model matches well with the fact that individuals at the bottom 0–20 quintile do not pay personal income taxes. However, the baseline model understates the tax payments of quintiles 20–40 and 40–60, and overstates the tax payments of the 80–100 quintile. Although the model matches the share of taxes paid by the income rich 90–99 individuals, it overstates the tax payments of the top 1% income rich (35% in the model and 21% in the data). In summary, the baseline model reproduces well the shares of taxes paid by most of the population, although it overstates the share of personal income taxes paid by the income richest individuals (the top 1% of the population).

**Table 11:** Share of personal income tax payments across the income distribution

	Quintiles					Top			Bottom
	0–20	20–40	40–60	60–80	80–100	90–95	95–99	99–100	0–10
Data	$-$ 0.2	0.7	7.0	19.4	73.2	13.8	20.6	21.0	0.0
Baseline	$-$ 0.2	$-$ 0.3	2.1	11.3	87.1	15.1	22.7	35.6	$-$ 0.09

Data source: Guner et al. (2020)

## Quantitative experiments: tax reforms

In this section, I quantify the macroeconomic and distributive effects of alternative tax reforms aimed at increasing the overall tax revenue of the government. The first reform increases the tax rates on labor income by shifting up the average tax function represented in Fig. 3. The second tax reform increases the degree of progressivity of the average tax function on labor income by increasing tax rates for income levels above the economy’s mean labor income. The third, fourth, and fifth reforms increase the effective tax rate on interest income, wealth, and inheritances, respectively. The last tax reform increases the effective tax rate on consumption. In all tax reforms, I assume that the government expenditure *G* adjusts so that the government’s budget balances in each period. This implies that an increase in total tax revenue translates into an equal increase in *G*.

### Increasing average tax rates on labor income

First, I explore reforms of the personal income tax that increase the average tax rate across the income distribution and maintain the degree of progressivity as in the baseline economy. In particular, I reduce the value of the parameter $\theta$ of the average tax function while keeping constant both the degree of progressivity of the tax function $\tau =0.1581$ and the level of income exempt from taxation (49% of average income). Figure 3 plots the average tax rate as a function of multiples of labor income for different levels of $\theta$. For instance, for $\theta =0.8919$, as in the baseline model, the average effective tax rate is 11% for income equal to mean income and 30% for income equal to 4.5 times mean income (99th percentile of the income distribution). If instead $\theta =0.75$, these average tax rates are 24% and 41%, respectively.

**Fig. 4:**
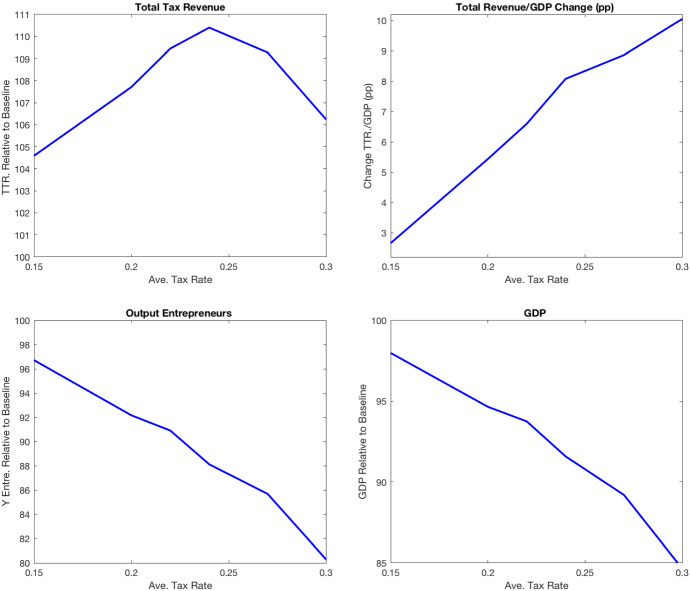
Increasing average tax rates on labor income

I compute the stationary equilibria implied by values of $\theta$ between 0.70 and 0.8919, where the latter corresponds to the baseline economy. For each of these economies, I compute the total tax revenue, fiscal pressure, and output and plot them in Fig. 4. The first panel of the figure shows that the government’s total revenue is maximized when $\theta =0.75$, which generates a substantial increase in total tax revenue (10%) and fiscal pressure (8 p.p.). The tax reform increases tax rates across income levels above the fixed income threshold. Indeed, the average effective tax rate increases from 11% to 25% at the economy’s mean income level, and from 30% to 41% at the 99th percentile of the income distribution. In terms of effective marginal tax rates, the reform implies an increase from 0.25 to 0.37 at the mean income level and from 0.41 to 0.50 at the 99th. percentile income level. This large increase in marginal tax rates is costly in terms of output, as the third and fourth panels of Fig. 4 show.

Table 12 reports other aggregate statistics for a sample of four economies characterized by $\theta = \{0.85, 0.80, 0.75,0.70\}$. At the stationary equilibrium where tax revenue is maximized ($\theta =0.75$), aggregate capital is 14% lower, and aggregate labor is 5% lower relative to the baseline economy. As a result, aggregate output decreases by 8.4%, and the output of entrepreneurs decreases by 12% relative to the baseline economy. The table also reports the revenue collected from each of the tax instruments relative to the baseline economy. In all the economies considered, personal income taxation, payroll taxation, and consumption taxation are the most important sources of tax revenue. Thus, changes in any of these revenue categories have substantial effects on the total tax revenue of the government. Both revenue from consumption taxation (9.4% of GDP) and social security contributions (15% of GDP) decrease when $\theta$ is reduced relative to the baseline economy. In particular, the reform that maximizes total tax revenue implies that consumption tax revenue decreases by 19% and social security contributions decrease by 11%. This result highlights the importance of modeling all sources of tax revenue when quantifying the effects of tax reforms on government finances.

**Table 12:** Aggregate effects of increasing average tax rates on labor and business income

	Baseline	$\theta =0.85$	$\theta =0.80$	$\theta =0.75$	$\theta =0.70$
Wage	100	99	98	97	95.7
r	100	107	117	127	137.5
GDP	100	98	94.6	91.6	84.6
Y entrep.	100	96.7	92	88	80
K	100	96.6	91	86	78
K entrep.	100	94	86	79	68.5
L	100	99	97	95	89
Total Tax Rev.	100	104.6	107.7	110	106
$\Delta$Total Tax Rev./GDP (pp)	0	2.6	5.4	8.1	10
PI Tax Rev.	100	121	140	157	163
SS Cont.	100	97	93	89	78
Consump. Tax	100	94	87	81	72
Corp. Tax	100	104	108	112	110.7
Wealth Tax	100	91	80	69	56
Inheritance Tax	100	96	90	84	73
PI Tax payments Quintiles (%)					
40–60	2.1	4.0	2.6	1.14	-0.23
60–80	11.3	14.3	17.1	18.7	12.7
80–100	87.1	82.1	80.6	80.4	87.8
Gini after-tax income	0.38	0.37	0.36	0.35	0.34
Gini consumption	0.32	0.31	0.306	0.29	0.28
Share wealth top 1%	21%	20.5%	19.7%	19%	18%

The increase in tax rates on business income raises the opportunity cost of entrepreneurship and the fraction of entrepreneurs decreases by 3.4%. Entrepreneurs hire 8.6% less labor and 20% less capital, which implies a reduction in the production of entrepreneurs of 12%.

The reform that maximizes the total tax revenue reduces the inequality in consumption even though it implies a higher inequality of before-tax income. Table 12 shows that the Gini coefficient of consumption decreases from 0.32 in the baseline to 0.29 and the Gini coefficient of after-tax income also decreases from 0.37 to 0.35. The positive impact of this reform on inequality comes from keeping constant both the progressivity parameter $\tau$ and the exempt income level of the labor income tax function. Also, the reform induces a reduction in the Gini coefficient of wealth induced by the decrease in entrepreneurship and capital accumulation. The share of wealth held by the top 1% richest decreases from 21 to 19%.

In summary, I find that it is possible to increase the total government revenue up to 10% and fiscal pressure by 8 percentage points by shifting up the average tax function so that the average tax rate roughly doubles (from 11 to 24%) at the mean income level. This reform implies that the average tax rate increases for all income levels above the exempt income level. In particular, at the exempt level of income (12,000 euros in 2015), the average tax rate increases from 0 to 15%, at the 90 income percentile (41,699 euros in 2015), the average tax rate increases from 18 to 32%, and at the 99 income percentile (94,974 euros in 2015), the average tax rate increases from 28 to 40%. As a result, the distribution of personal income tax payments shifts from the top 80–100 quintiles (incomes above 34,000 euros in 2015) to the 60–80 quintiles (incomes between 22,673 and 34,000 euros in 2015) (see Table 12). Next, I explore tax reforms targeted to increase tax revenue from income-rich households.

### Increasing the degree of progressivity of the labor income tax function

In a second set of experiments, I follow (Guner et al. 2020) to consider reforms that increase the degree of progressivity of the labor income average tax function while keeping the average tax rate constant (tax rate applied to the mean income level). In particular, I change the value of the parameter $\tau$ of the average tax function (Eq. 3) and keep all other parameters at the values calibrated in the baseline model. As $\tau$ increases, the average tax rate increases for levels of income above the mean income in the economy.^12^ Since the baseline model matches the top tail of the income and wealth distributions, it provides an interesting framework to quantify the extra revenue obtained by increasing tax rates to income-rich households.

I find that government’s total tax revenue is maximized when $\tau =0.16$, but total tax revenue only increases by 0.01%. Although the revenue from personal income taxes increases slightly, the revenue from other taxes decreases. In Table 13, I report the effects of changing $\tau$ on the different sources of tax revenue. I find that the revenue from personal income taxes is maximized when $\tau =0.23$. This reform increases the revenue from personal income taxes by 4.5%, but total tax revenue decreases by 1% due to the decreases in the revenue from social security contributions (3.6%) and consumption taxes (5%). Increasing the progressivity parameter implies higher tax rates for households with labor income above the economy’s mean level. This discourages the labor supply of workers ($-1.5\%$), accumulation of capital by entrepreneurs ($-12.6\%$), and the overall output of the economy ($-4.2\%$). These findings are consistent with those of Guner et al. (2020) in a model without entrepreneurs. They find that $\tau =0.19$ maximizes revenue from personal income taxes, while total tax revenue decreases by 1.5%.

**Table 13:** Aggregate effects of progressivity of the labor income tax function ($\tau$)

	$\tau =0$	$\tau =0.10$	$\tau =0.16$	$\tau =0.23$	$\tau =0.26$
GDP	110.1	103.5	99.3	95.8	93.7
Y entrep.	115	105.8	99.	93.8	91
K	126.7	108	98.5	91.5	88
K entrep.	139	113	97.8	87.4	82
L	102.3	101	99.7	98.5	97.3
Total Tax Rev.	94	99.3	100.01	98.9	97.3
$\Delta$Total Tax Rev./GDP	$-$ 6pp	$-$ 1.6pp	0.26pp	1.2pp	1.5pp
PI Tax Rev.	59.3	92	100.2	104.5	103.5
SS contrib.	109.4	102.4	99.5	96.4	94.5
Consump Tax	115	104	99.3	95	93

In sum, the total tax revenue cannot increase by just increasing the progressivity parameter of the average tax function.^13^ Such reform is effective though in reducing the inequality of the distribution of consumption, since the Gini coefficient decreases from 0.32 in the baseline economy to 0.29 in the economy where $\tau =0.23$.

### Capital income, wealth, and inheritance taxation

In the previous experiments, I considered reforms that increase tax rates on labor and business income. In this section, I consider tax reforms that increase tax rates for wealth-rich individuals. The first set of reforms increase tax rates on capital income of households (interest income). The second set of experiments increase tax rates on wealth and the third increase tax rates on inheritances.

#### Increase in tax rates on capital income

**Fig. 5:**
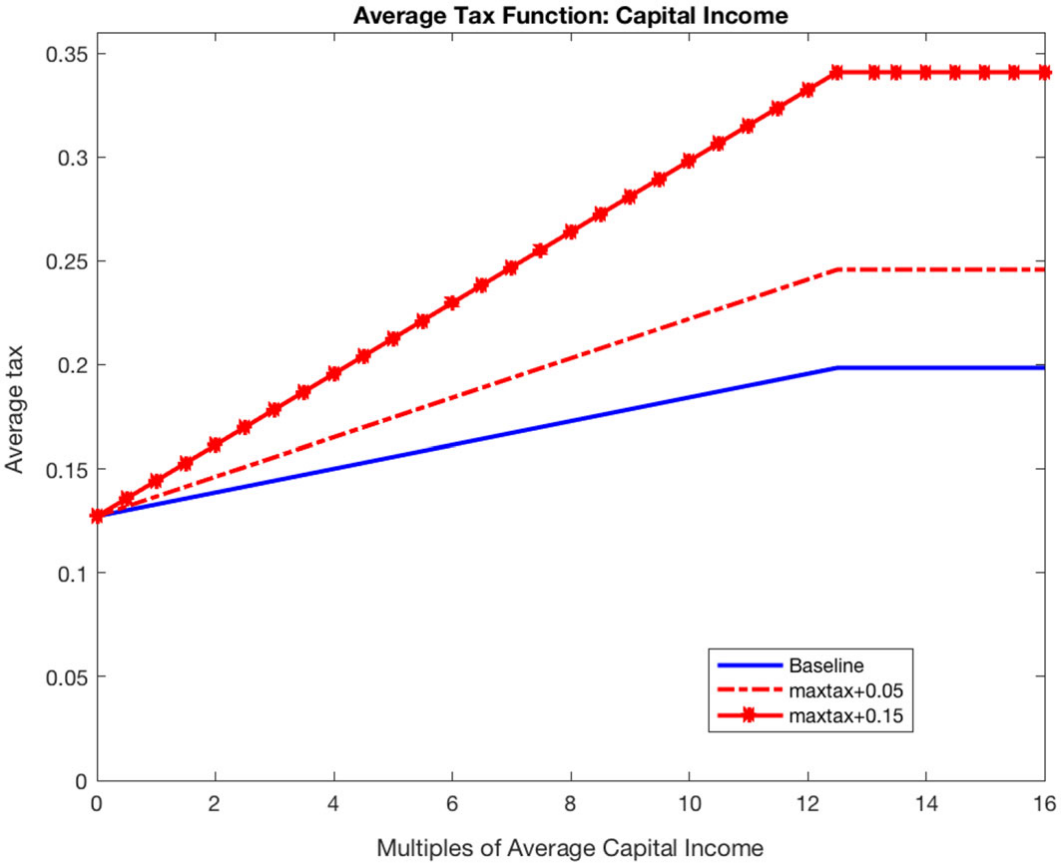
Average tax on capital income function

I consider tax reforms that increase the marginal tax rate for all levels of capital income by changing the parameter $\eta _1$ of the average tax function (4). In all tax reforms, the threshold level of income at which the maximum marginal tax applies is kept constant (it equals 13.14 times the average capital income of households in the economy). Figure 5 represents the average tax rates at multiples of average capital income in the baseline economy ($\eta _1=0.0057$) and in two alternative economies characterized by $\eta _1=\{0.0098,0.017\}$. These reforms increase the average tax rate across the distribution of capital income and imply a maximum tax rate of 0.25 and 0.35, while it is 0.20 in the baseline economy.^14^

**Fig. 6:**
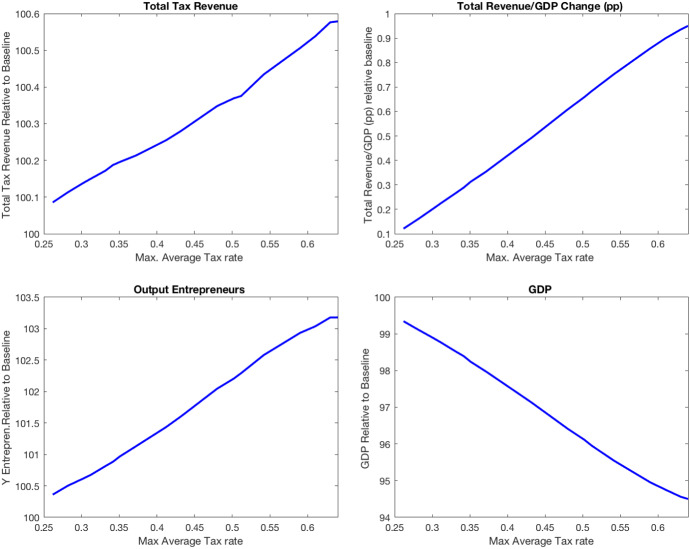
Increasing tax rates on capital income

I find that all the reforms considered imply a small increase in total tax revenue. While the revenue from personal income tax increases, the revenue from social security contributions and consumption taxation decrease in all reforms. The first panel of Fig. 6 plots total tax revenue (relative to the baseline economy) in each of the reforms. For instance, the reform that implies doubling the maximum average tax rate (from 0.20 to 0.40) increases total tax revenue by 0.25%. While the revenue from personal income taxes increases by 2.2%, social security contributions decrease by 1%, and revenue from consumption taxes decreases by 0.7%. The decrease in tax revenue is explained by the reduction in GDP by 2.6% (see the lower right panel of Fig. 6). As a result of the increase in overall tax revenue and the decrease in GDP, the fiscal pressure statistic shows an increase of 0.44 percentage points.^15^

The reform has interesting effects on entrepreneurship, which are consistent with the findings in Kitao (2008). Although the reform increases the interest rate, the opportunity cost of being an entrepreneur decreases because of the increase in the average tax rate on capital income. As a result, the number of entrepreneurs increases by 1.7% and output of entrepreneurs increases by 1.4% (see Fig. 6).

#### Increase in tax rates on wealth

**Fig. 7:**
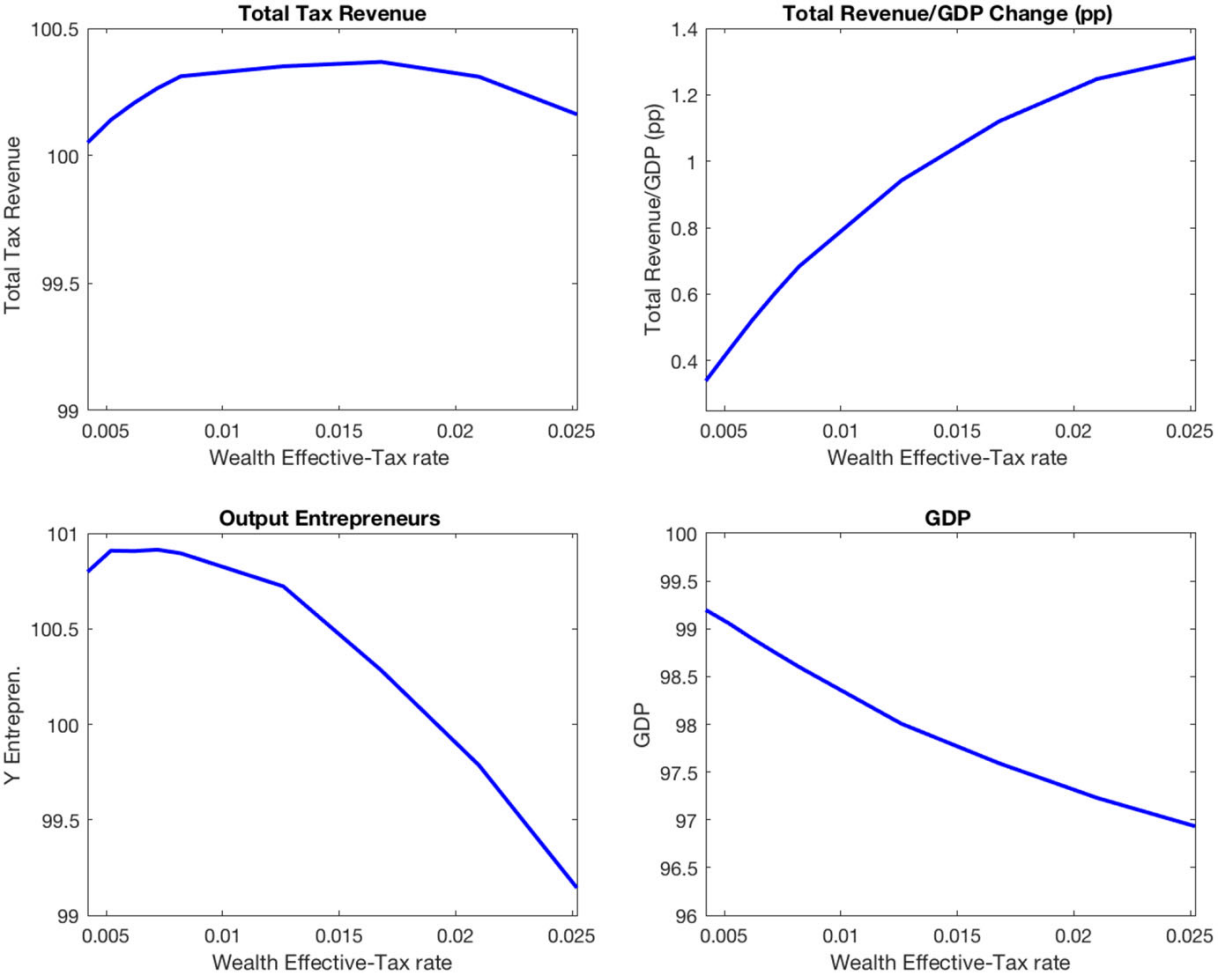
Increasing tax rate on wealth at the top 5% of distribution

In the baseline economy, wealth is taxed at a flat rate of 0.42% above the exempt level of wealth (30 times the average individual income). In this section, I quantify the effects of reducing the exempt wealth and increasing the effective tax rate. In particular, in all reforms considered, the exempt wealth equals 10 times the average individual income (about 233,333 euros in 2015). Figure 7 illustrates the main effects of the simulated reforms of wealth taxation characterized by an effective tax rate between 0.42% and 2%.

I find that total tax revenue is maximized when the effective tax rate on wealth is 1.68%, but it increases only by 0.4%. The large increase in the revenue from wealth taxation (358%) is almost canceled out by the decrease in the revenue from personal income taxes ($-2$%) and social security contributions ($-2$%). I also find that this reform has large negative effects on capital accumulation ($-7.2$%) and GDP ($-2.4$%). As emphasized by Guvenen et al. (2019), taxing wealth reduces the opportunity cost of entrepreneurship inducing wealthy households with high entrepreneurial to be entrepreneurs. As a result, the number of entrepreneurs increases by 4%. Although the capital employed by entrepreneurs decreases by 5%, the output of entrepreneurs increases by 0.3%.

#### Increase in taxation of inheritances

**Fig. 8:**
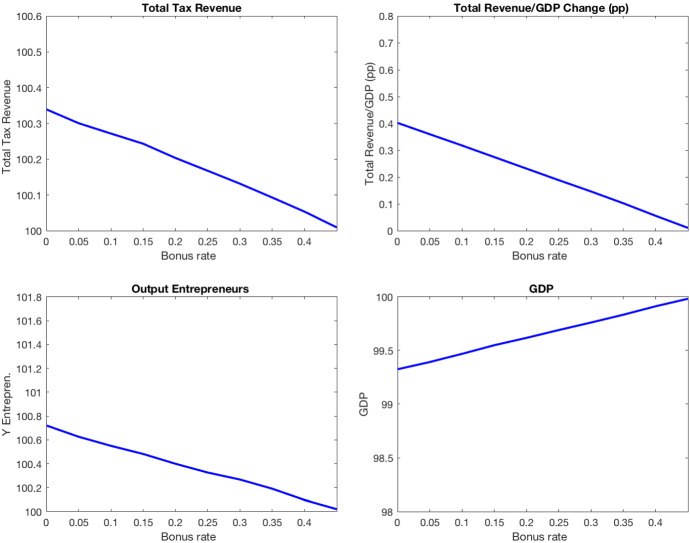
Increasing taxation of inheritances

In the baseline economy, inheritances are taxed according to the statutory tax schedule (see Table 5) and a bonus rate of 46.2%. In this experiment, I quantify the effects of increasing the revenue from inheritance taxation by decreasing the bonus rate used to compute the inheritance tax quota. Figure 8 plots the overall tax revenue, fiscal pressure, GDP, and output of entrepreneurs in economies characterized by a bonus rate between 46.2% and 0%.

I find that the elimination of the bonus rate increases the total tax revenue by 0.7%. Although the increase in the revenue from inheritance taxes is large (73%), it is compensated by the decreases in social security contributions ($-1\%$) and the revenue from consumption taxes ($-1\%$). Moreover, the reform induces a reduction in aggregate capital by 3% and GDP by 1%. Interestingly, the output of entrepreneurs increases by 0.7%.

In summary, increasing taxes on wealth-rich individuals through capital income taxes, wealth taxes, or inheritance taxes have relatively small effects on the overall tax revenue and fiscal pressure as the summary in Table 14 shows.

**Table 14:** Reforms that increase taxes to rich households

	PI: progressivity	Capital income	Wealth	Inheritances
Total Tax Revenue	100.005	100.6	100.4	100.34
$\Delta$ TTR/GDP (pp)	0.04	0.95	1.12	0.4
$\Delta GDP$	$-$ 0.1%	$-$ 1.8%	$-$ 2.4%	$-$ 0.7%
$\Delta Y_e$	$-$ 0.1%	3.2%	0.3%	0.7%
$\Delta$Gini Cons.	0.0	$-$ 0.06	$-$ 0.01	0.0

The output of entrepreneurs increases when tax rates on capital income, wealth, and inheritances increase. Taxing capital income reduces the opportunity cost of entrepreneurial investment and positively affects entrepreneurial output (Kitao 2008). Similarly, increasing tax rates on wealth relative to tax rates on business income shifts the tax burden toward the unproductive business, which positively affects aggregate production (Guvenen et al. 2019).

### Increase in the consumption tax rate

In this section, I quantify the effects of increasing the consumption tax rate on the government’s total tax revenue. Increasing the consumption tax reduces the value of wealth in terms of consumption. The burden of consumption taxation falls importantly on retirees because of two reasons. First, retirees’ marginal propensity to consume is relatively large. Second, they hold a large fraction of the wealth in Spain. Indeed, Table 15 shows that among the top 1% richest households, retirees are the largest fraction (46%) relative to entrepreneurs (36%) and workers (18%). Interestingly, in the US economy, only 20% of the top 1% wealth-rich households are retirees.^16^

**Table 15:** Occupations at the top 1% of the wealth distribution

	Spain		US	
	Top 1%	Top 5%	Top 1%	Top 5%
Entrepreneurs	36	27	51	39
Workers	18	20	28	35
Retirees	46	51	20	26

Data source: EFF 2017 (Spain) and SCF 2019 (US)

I compute the stationary equilibria of economies in which the effective tax rate on consumption takes values between 0.15 (baseline economy) and 0.30. Table 16 illustrates the main findings of these experiments. I find that increasing the consumption tax rate is a very effective tool to increase the government’s revenue. An increase in 7 p.p. of the consumption tax rate implies that overall tax revenue increases by 9% and fiscal pressure by 3.6 p.p. With a tax rate of 30%, the fiscal pressure increases by 7 p.p. which is the gap between Spain and the average of the EU in 2021.

In the long-run, aggregate capital, labor, and GDP are not affected by increasing the consumption tax rate. Increasing the tax on consumption does not affect the labor supply decision because the income and substitution effects cancel out in this model.^17^ I also find that the Gini coefficient of consumption is the same across stationary equilibria.

**Table 16:** Increasing consumption taxation

	$\tau _c=0.15$	$\tau _c=0.22$	$\tau _c=0.25$	$\tau _c=0.30$
Total tax rev.	100	109.1	112.6	118.2
$\Delta$total tax rev./GDP (pp)	0	3.6	5.0	7.2
$\Delta$ rev. consumption tax	100	138.2	153.3	177.0

In summary, among all the reforms considered, increasing taxes on consumption is the most effective in terms of raising tax revenue because it does not affect output in the economy. However, reforms that increase the tax rate on consumption do not reduce inequality measures. If policymakers care about reducing consumption inequality, they should consider increasing the average tax on income instead.

## Conclusions

I consider the macroeconomic and redistributive effects of tax reforms aimed at increasing tax revenue in Spain. I find two reforms that raise fiscal pressure in Spain to the average value among countries in the Euro area. The first reform involves doubling the average effective tax rate on personal income (labor and business income) for all individuals whose income is above a (fixed) threshold level. I find that this reform reduces the inequality in after-tax income, wealth, and consumption. However, it implies a substantial GDP reduction. The second reform increases the flat tax rate on consumption by fifteen percentage points. While this reform does not reduce long-run output, it does not decrease household inequality. All in all, the desirability of the two reforms depends on the government’s preferences for reducing inequality at the expense of aggregate output losses.

In my investigation, I take as given the need to raise tax revenue. Of course, an alternative might be not to increase taxes which should necessarily lead to a reduction in the welfare state in Spain. Similarly, my analysis has not assessed the support for alternative tax reforms, which would require computing the transitional dynamics and taking a stand on how the tax revenue is spent.^18^ These important issues are beyond the scope of this paper.

## A Appendix: consumption taxation in an Aiyagari–Huggett model

Consider an Aiyagari–Huggett model economy with endogenous labor supply and a consumption tax. The state of the household is the tuple (*a*, *z*) where *a* denotes the initial assets and *z* the current realization of the labor productivity shock. The labor productivity shock is persistent. I denote by $(a',z')$ the next period assets and realization of the labor productivity shock. The Bellman equation is:

$$\begin{aligned} v(a,z)&=\max _{c,h,a'}\{ \ln (c) -\phi \frac{h^{1-\sigma }}{1-\sigma } +\beta E[v(a',z')/z]\} \\&\quad \text {subject to:} \\&\quad (1+\tau _c)c+a'= zwh +(1+r)a \\&\quad a'\ge 0 \end{aligned}$$

where c denotes consumption, h denotes working time, and r is the return of a safe asset. Below, I show that a consumption tax does not affect labor supply and savings in this model. The Lagrangian function is:

$$\begin{aligned} L(c,h,a',\lambda )= & {} \ln (c)-\phi \frac{h^{1-\sigma }}{1-\sigma }\nonumber \\{} & {} \quad +\beta E[v(a',z')/z]+\lambda [ zwh +(1+r)a-(1+\tau _c)c-a']+\mu a' \end{aligned}$$

The FOC imply:

$$\begin{aligned}&c: \frac{1}{c} -\lambda (1+\tau _c)=0\\&h: -\phi h^{-\sigma }+\lambda zw=0 \\&a': \beta E[v'(a',z')/z]-\lambda +\mu =0 \\&KT: a' \mu =0, \mu \ge 0, a' \ge 0 \\&\lambda : (1+\tau _c)c+a'=zwh +(1+r)a \end{aligned}$$

where $\lambda$ is the multiplier associated with the budget constraint. The Envelope condition yields:

$$\begin{aligned} v'(a,z)=\lambda (1+r) \end{aligned}$$

Optimal labor supply, savings, and consumption are characterized by: The FOC imply:

$$\begin{aligned}&\frac{1}{(1+\tau _c)c}\le \beta E[v'(a',z')/z] =\frac{1}{(1+\tau _c)c'}(1+r) \text {with = if } a'=0 \\&\phi h^{-\sigma }= \frac{zw}{(1+\tau _c)c} \\&(1+\tau _c)c+a'=zwh +(1+r)a \end{aligned}$$

The above FOCs define the optimal policies of consumption, hours, and savings, which I write as follows:

$$\begin{aligned}&\frac{1}{(1+\tau _c)c(a,z;\tau _c)}\le \frac{1}{(1+\tau _c)c(a',z';\tau _c)}(1+r) \text {with = if } a'(a,z;\tau _c)=0 \\&\phi {h(a,z;\tau _c)}^{-\sigma }= \frac{zw}{(1+\tau _c)c(a,z;\tau _c)} \\&(1+\tau _c)c(a,z;\tau _c)+a'(a,z;\tau _c)=zw h(a,z;\tau _c) +(1+r)a \end{aligned}$$

Guess that

$$\begin{aligned}&c(a,z;0)=(1+\tau _c)c(a,z;\tau _c)\\&h(a,z;0)=h(a,z;\tau _c)\\&a'(a,z;0)=a'(a,z;\tau _c) \end{aligned}$$

Substituting in the FOC characterizing optimal policies, it is easy to see that the FOC are satisfied. I thus conclude that the guess is correct. It follows that the consumption tax does not affect hours of work and savings decisions.

## Footnotes

1 See Plan de Recuperación, Transformación y Resilencia, Componente 28, pages 5–9, 2021.

2 Allub and Erosa (2019) show that a theory with shocks to entrepreneurial and worker abilities accounts well for the distribution of income across and within occupations in Brazil.

3 Gouveia and Strauss (1994), and Guner et al. (2014) provide estimates of effective tax rates for the USA, and Calonge and Conesa (2003) and García-Miralles et al. (2019) for estimates for Spain.

4 Guvenen et al. (2019) compare the macroeconomic and welfare effects of wealth taxation and capital income taxation in a model of heterogeneous investment returns. In a similar framework, (Macnamara et al. 2022) estimate the response of income to marginal tax rates in the US economy.

5 Other papers quantifying the effect of increasing labor income tax progressivity or increasing the top marginal tax rates on the government’s revenue include (Badel et al. 2020) and (Kindermann and Krueger 2022).

6 This utility function implies that the Frisch elasticity of labor supply equals $\frac{1}{\gamma }$.

7 From now on, when I refer to labor income, I mean labor income and business (or self-employment) income.

8 The level of maximum monthly earnings taxed was 3600 euros in 2015, which was about 1.75 times the mean monthly earnings in Spain.

9 Adult population is defined as the number of individuals older than 24.

10 In the baseline calibration, $rr(z_w)= \{0.09,0.13,0.2,0.3,0.4,0.6,0.8,1.3,1.9,2.8 \}$ gives the best fit of the distribution of pensions.

11 Source: Agencia Tributaria, ‘Tablas evolutivas: Variables Tributarias y Tipos Impositivos,’ available online at sede.agenciatributaria.gob.es.

12 The average tax rate (0.11), as well as the exempt income level (49% of the average income of the economy) stay constant.

13 I have also experimented with reforms that increase the marginal tax rate to the top 5% income-rich, but the implied increase in total tax revenue is negligible.

14 The maximum tax rate applies to the 99 percentile of the distribution of capital income.

15 I also considered a reform that eliminates the difference between the tax schedules on capital income and labor income. In particular, I used the average tax function $T_l(\cdot )$ to compute average tax payments on overall household income (the sum of labor income and capital income). I found that this reform *reduces* total tax revenue by 0.65%. The increase in tax rates on capital income induces a decrease in the aggregate capital stock (1.6%) and a decrease in output (0.6%).

16 I thank Anastasis Kokufakis for providing these statistics from the SCF.

17 This result is shown analytically in Appendix A for a version of this model without occupational choice. Across the stationary equilibria characterized by a given tax rate on consumption, households’ policy functions of assets and labor supply are identical as well as after-tax optimal consumption.

18 For instance, in a dynastic model calibrated to the US economy, Fuster et al. 2008 find that eliminating income taxation and increasing the tax rate on consumption benefits 75% of the population living at the moment of the reform.
